# Carrot RG-I Reduces Interindividual Differences between 24 Adults through Consistent Effects on Gut Microbiota Composition and Function Ex Vivo

**DOI:** 10.3390/nu15092090

**Published:** 2023-04-26

**Authors:** Pieter Van den Abbeele, Stef Deyaert, Ruud Albers, Aurélien Baudot, Annick Mercenier

**Affiliations:** 1 Cryptobiotix SA, 9052 Ghent, Belgium; pieter.vandenabbeele@cryptobiotix.eu (P.V.d.A.); stef.deyaert@cryptobiotix.eu (S.D.); aurelien.baudot@cryptobiotix.eu (A.B.); 2 Nutrileads BV, 6708 WH Wageningen, The Netherlands; ruud.albers@nutrileads.com

**Keywords:** pectin, rhamnogalacturonan I, *Bifidobacteriaceae*, *Bacteroidaceae*, acetate, propionate, butyrate, ex vivo, prebiotic

## Abstract

The human gut microbiota is characterized by large interpersonal differences, which are not only linked to health and disease but also determine the outcome of nutritional interventions. In line with the growing interest for developing targeted gut microbiota modulators, the selectivity of a carrot-derived rhamnogalacturonan I (cRG-I) was compared to substrates with demonstrated low (inulin, IN) and high selectivity (xanthan, XA), at a human equivalent dose (HED) of 1.5 g/d. The high throughput of the ex vivo SIFR^®^ technology, validated to generate predictive insights for clinical findings, enabled the inclusion of 24 human adults. Such an unprecedented high number of samples in the context of in vitro gut microbiota modelling allowed a coverage of clinically relevant interpersonal differences in gut microbiota composition and function. A key finding was that cRG-I supplementation (already at an HED of 0.3 g/d) lowered interpersonal compositional differences due to the selective stimulation of taxa that were consistently present among human adults, including OTUs related to *Bacteroides dorei/vulgatus* and *Bifidobacterium longum* (suspected keystone species)*, Bacteroides thetaiotaomicron*, *Bifidobacterium adolescentis* and butyrate-producing taxa such as *Blautia* sp., *Anaerobutyricum hallii,* and *Faecalibacterium prausnitzii*. In contrast, both IN and XA treatments increased interpersonal compositional differences. For IN, this followed from its low specificity. For XA, it was rather the extremely high selectivity of XA fermentation that caused large differences between 15 responders and 9 nonresponders, caused by the presence/absence of highly specific XA-fermenting taxa. While all test compounds significantly enhanced acetate, propionate, butyrate, and gas production, cRG-I resulted in a significantly higher acetate (+40%), propionate (+22%), yet a lower gas production (–44%) compared to IN. cRG-I could thus result in overall more robust beneficial effects, while also being better tolerated. Moreover, owing to its remarkable homogenization effect on microbial composition and metabolite production, cRG-I could lead to more predictable outcomes compared to substrates that are less specific or overly specific.

## 1. Introduction

Over the past decades, the human gut microbiota has been related with human health and, when aberrant, to metabolic disorders (obesity, type 2 diabetes, nonalcoholic liver disease, cardiometabolic diseases) [1], inflammatory bowel diseases [2,3], cancer [4], celiac disease [5], and even brain-related conditions [6]. Numerous molecular mechanisms have been unravelled to explain how gut microbes could protect from or contribute to diseases as reviewed by de Vos et al. (2022) [7]. Amongst a broad range of microbial metabolites, short-chain fatty acids (SCFA; mainly acetate, propionate, and butyrate), main end-metabolites of the colonic fermentation of glycans, have been ascribed potent health benefits [8,9]. SCFA production results from complex interactions between a broad range of gut microbes [10,11]. As recently demonstrated by Lavelle et al., the composition of the gut microbiota and the prevalence of keystone species is, however, prone to major interpersonal differences [12]. These interpersonal differences are clinically relevant as they impact the outcome of interventions [13,14]. They are, for instance, recognized as a key contributor for the inconsistent reports on the benefits of fibre intake [15]. In an attempt to stratify human subjects based on gut microbiota composition, the concept of enterotypes has been introduced [16]. While the exact classification of gut microbiota in distinct gut enterotypes is still evolving [17,18], it is clear that *Prevotella*, *Bacteroides* and *Ruminococcus* are three main contributors to the microbiota variation in healthy subjects [19]. Even if the health relevance of enterotypes remains to be elucidated, they are a useful stratification tool in gut microbiota research.

**Figure 1:**
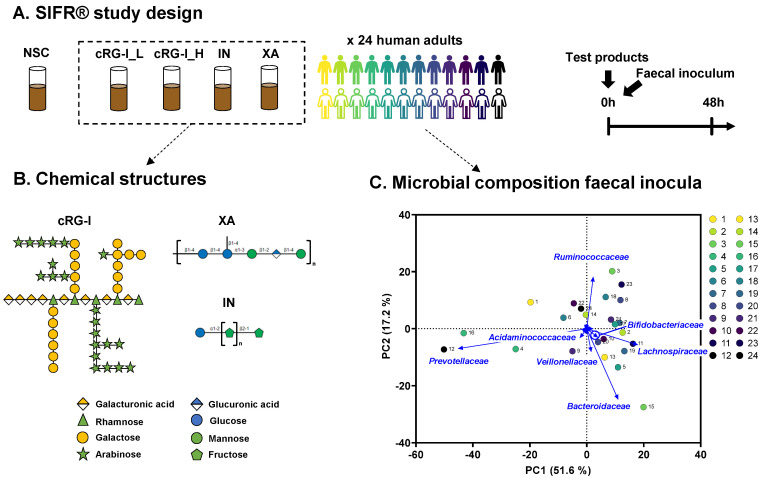
**Study design using the ex vivo SIFR^®^ technology to assess the selectivity by which cRG-I, IN, and XA affect the gut microbiota of 24 human adults that together covered clinically relevant interpersonal differences in microbiota composition**. (**A**) Reactor design using the ex vivo SIFR^®^ technology to test the impact of cRG-I, IN, and XA at an equivalent dose of 0.3 g/d (cRG-I_L) or 1.5 g/d (cRG-I_H, IN and XA), compared to a reference without additional substrate (NSC) in faecal samples of 24 human adults. (**B**) Schematic chemical structures of the different test products and (**C**) PCA biplot based on centred values of microbial families (%) at baseline (0 h), as part of the faecal microbiota for each of the 24 human adults. SIFR = systemic intestinal fermentation research; cRG-I = carrot-derived rhamnogalacturonan; IN = inulin; XA = xanthan; NSC = no-substrate control.

Amongst strategies that aim to improve human health by targeting the gut microbiota, dietary fibres such as pectin-derived polysaccharides [20] and prebiotics are gaining increasing interest [21,22]. Despite being defined as substrates that are selectively utilized by host microorganisms (thus conferring a health benefit) [23], recent reports describe the low selectivity by which several traditional prebiotics impact the human gut microbiota [24,25]; e.g., inulin (IN) is a low specificity fibre that can be used by a wide array of gut microbes [24,25], likely due to its relatively simple structure (Figure 1B), high solubility, and historical abundance in the diet. Such low selectivity results in unpredictable effects, which are determined by the baseline gut microbial communities present within each individual. An excessively high specificity as, for instance, found for xanthan (XA), which has a more complex structure (Figure 1B) and has only recently been introduced to the human diet, is also not desirable as it can only be used by a very narrow range of bacteria which are either present or absent in the microbiota, leading to responder and nonresponders [26]. There is thus a growing interest in developing prebiotics or dietary fibres with the desired level of specificity, i.e., that are selectively fermented by commonly present beneficial commensal microorganisms, thus minimizing interpersonal differences and causing more predictable outcomes of interventions [14,24,25]. A potential candidate with the desired level of specificity is carrot-derived rhamnogalacturonan-I (cRG-I), which, like XA, possesses a complex structure (Figure 1B). Moreover, cRG-I has been shown to be fermented by specific, commonly present taxa during in vitro studies [27,28]. These studies however only tested the fermentation of cRG-I for one [27] or a limited number of donors (n = 4) [28], thus limiting strong conclusions on the tentative selectivity by which cRG-I impacts the human gut microbiota.

While a large number of test subjects is indubitably necessary to obtain representative insights in the human gut microbiota, it is particularly important when the aim is to assess the tentative selectivity by which a substrate affects the microbiota that can greatly differ among human subjects. In this context, a main disadvantage of the commonly used in vitro models is their low throughput. Moreover, current in vitro models may also be biased by substantial alterations between the in vivo derived microbiota and the microbiota that is establishing itself in the laboratory systems, both for short-term [27,29,30,31] and long-term models [32,33,34]. Recently, the ex vivo SIFR^®^ technology (Systemic Intestinal Fermentation Research; pronounced “*cipher*”), a high-throughput, bioreactor-based gut model, was shown to generate, within 24–48 h of incubation, insights down to species level that are predictive for outcomes of clinical studies where prebiotics are repeatedly dosed over 2–6 weeks [35].

In this study, the SIFR^®^ technology was used to assess the selectivity by which cRG-I affects the composition and metabolite production of the human adult gut microbiota. The high throughput of the ex vivo SIFR^®^ technology enabled the inclusion of 24 human adults in the study design, which was crucial for addressing this central hypothesis. The specificity of cRG-I was compared with IN and XA as examples of low-specificity and very high-specificity fibres.

## 2. Materials and Methods

### 2.1. Test Compounds

The test compounds evaluated were IN from chicory (I2255, Merck, Overijse, Belgium), XA (3557, Carl Roth, Karlsruhe, Germany), and cRG-I (BeniCaros^®^, Nutrileads, Wageningen, The Netherlands). Inulin is a polymer of β(2,1)-bond-linked fructose residues with a chain-terminating glucose with a fructose:glucose ratio of 20:1. Xanthan is constituted of repeated pentasaccharides of β(1,4) glucose moieties decorated with trisaccharide side chains comprising 2 mannose and 1 glucuronic acid units. cRG-I is a polydisperse pectic polysaccharide derived from carrot pomace and highly enriched (80%) in the RG-I domain. The monosaccharide composition of cRG-I is (% mol/mol): rhamnose, 14.3; arabinose, 34.8; galactose, 19.6; fucose, 0.8; glucose, 4.3; mannose, 0.9; xylose, 0.7; galacturonic acid, 25 (25). The schematic structures of the compounds are illustrated in Figure 1B.

### 2.2. SIFR^®^ Technology

The SIFR^®^ technology was developed to study the human gut microbiota in a highly biorelevant manner across numerous parallel test conditions (both treatments and test subjects) [35]. Briefly, individual bioreactors were processed in parallel in a bioreactor management device (Cryptobiotix, Ghent, Belgium). Each bioreactor contained 5 mL of a nutritional medium–faecal inoculum blend supplemented with 1.5 g test compound/L (also 0.3 g/L for cRG-I), then sealed individually, before being rendered anaerobic. Blend M0003 was used for the preparation of the nutritional medium (Cryptobiotix, Ghent, Belgium). After preparation, bioreactors were incubated under continuous agitation (140 rpm) at 37 °C (MaxQ 6000, Thermo Scientific, Thermo Fisher Scientific, Merelbeke, Belgium).

Five experimental conditions were tested for 24 human adults: a no-substrate control (NSC), a 0.3 g/d low dose of cRG-I (cRG-I_L), a 1.5 g/d high dose of cRG-I (cRG-I_H), 1.5 g/d inulin (IN), and 1.5 g/d of xanthan (XA) (Figure 1A). For each of the 24 faecal samples, an NSC incubation was initiated simultaneously, consisting of an optimized nutritional medium and microbiota without test product. The advantage of comparing test products to such NSC is that any changes between the NSC and test products can solely be attributed to the addition of the test products. Following a 48 h incubation, the pressure was measured in the bioreactors’ headspace, and liquid samples were subsequently collected for the analysis of key fermentation parameters and microbial composition and metabolites.

Fresh faecal samples were collected according to a procedure approved by the Ethical Committee of the University Hospital Ghent (reference number BC-09977). This procedure required participants to sign an informed consent in which they donated their faecal sample for the current study. The selection criteria for the 24 donor samples used herein were as follows: 25–65 years of age, no antibiotic use in the past 3 months, no gastrointestinal disorders (cancer, ulcers, IBD), no use of probiotic, nonsmoking, alcohol consumption <3 units/d and BMI < 30. For this specific study, 13 male and 11 female donor samples were assessed. The age of the test subjects ranged from 28 to 61 years and was on average 37.8 years (37.8 years for male subjects; 39.1 years for female subjects).

### 2.3. Fundamental Fermentation Parameters

SCFA (acetate, propionate, butyrate, and valerate) and branched-chain fatty acids (bCFA; sum of isobutyrate, isocaproate, and isovalerate) were extracted from the samples with diethyl ether, after addition of 2-methyl hexanoic acid as an internal standard. Briefly, 0.5 mL samples were diluted in distilled water (1:3), acidified with 0.5 mL of 48% sulfuric acid, after which an excess of sodium chloride was added along with 0.2 mL of internal standard (2-methylhexanoic acid) and 2 mL of diethyl ether. Upon homogenization and subsequent separation of the water and diethyl ether layer, diethyl ether extracts were collected and analysed using a Trace 1300 chromatograph (Thermo Fisher Scientific, Merelbeke, Belgium) equipped with a Stabilwax-DA capillary GC column, a flame ionization detector, and a split injector using nitrogen gas as the carrier and makeup gas. The injection volume was 1 µL and the temperature profile was set from 110 °C to 240 °C. The carrier gas was nitrogen, and the temperatures of the injector and detector were 240 and 250 °C, respectively. The sample pH was measured using an electrode (Hannah Instruments Edge HI2002, Temse, Belgium).

### 2.4. Microbiota Phylogenetic Analysis: Quantitative 16S rRNA Gene Profiling

Quantitative data were obtained by correcting abundances (%; 16S rRNA gene profiling) with total cell counts (cells/mL; flow cytometry), resulting in the estimated absolute cell counts per mL of different taxonomic groups. Initially, a bacterial cell pellet was obtained by the centrifugation of 1 mL sample for 5 min at 9000× *g*. DNA was extracted via the SPINeasy DNA Kit for Soil (MP Biomedicals, Eschwege, Germany), according to the manufacturer’s instructions. Subsequently, library preparation and sequencing were performed on an Illumina MiSeq platform with v3 chemistry. The 16S rRNA gene V3-V4 hypervariable regions were amplified using primers 341F (5’-CCT ACG GGN GGC WGC AG-3’) and 785Rmod (5’-GAC TAC HVG GGT ATC TAA KCC-3’). Results were analysed at different taxonomic levels (phylum, family, and OTU level). The α-diversity (species richness) was estimated via the Chao1 diversity index. As compared to other indices, this index estimates the number of missing OTUs (and thus “counts the uncountable”) [36]. Further, the β-diversity (dissimilarity between samples) was assessed via the weighted Unifrac index, which accounts for both relatedness and the abundance of taxa. For the total cell count analysis, liquid samples were diluted in anaerobic phosphate-buffered saline (PBS), after which cells were stained with SYTO 16 at a final concentration of 1µM and counted via a BD FACS Verse flow cytometer (BD, Erembodegem, Belgium). Data were analysed using FlowJo, version 10.8.1.

### 2.5. Statistical Analysis

All univariate and multivariate analyses were performed using GraphPad Prism (v9.3.1; www.graphpad.comm, accessed on 23 November 2022), while the regularized canonical correlation analysis (rCCA) was executed using the mixOmics package with the shrinkage method for the estimation of penalisation parameters (version 6.16.3) in R (4.1.1; www.r-project.org, accessed on 23 November 2022) [37]. Treatment effects were compared with the NSC using a repeated measures ANOVA (based on paired testing) and *p*-values were corrected with Benjamini–Hochberg’s method [38] (FDR = 0.05). Paired testing (repeated-measures ANOVA) was performed for setups considering 24 donors with n = 1. For the analysis of the microbial composition, three measures were taken. First, the aforementioned statistical analysis was performed on the log_10_-transformed values. Second, a value of a given taxonomic group below the limit of detection (LOD) was considered equal to the overall LOD according to the procedure elaborated by Van den Abbeele et al. (2023) [35]. Finally, a threshold was set to retain the 100 most abundant OTUs in the analysis, to avoid excessive *p*-values corrections.

## 3. Results

### 3.1. The Study Cohort Covered Established Interpersonal Differences in Enterotypes Described for Human Adult Gut Microbiota

At the family level, there were marked differences in faecal microbiota composition among the 24 human adults, mostly due to differences in *Prevotellaceae*, *Bacteroidaceae,* and/or *Ruminococcaceae* (Figure 1C). The stratification of faecal microbiota based on these families is in line with the classification of faecal microbiota according to the concept of enterotypes [16,17,18,19].

### 3.2. cRG-I Leads to the Most Marked and Consistent Effects on Microbiota Composition and Metabolite Production

**Figure 2:**
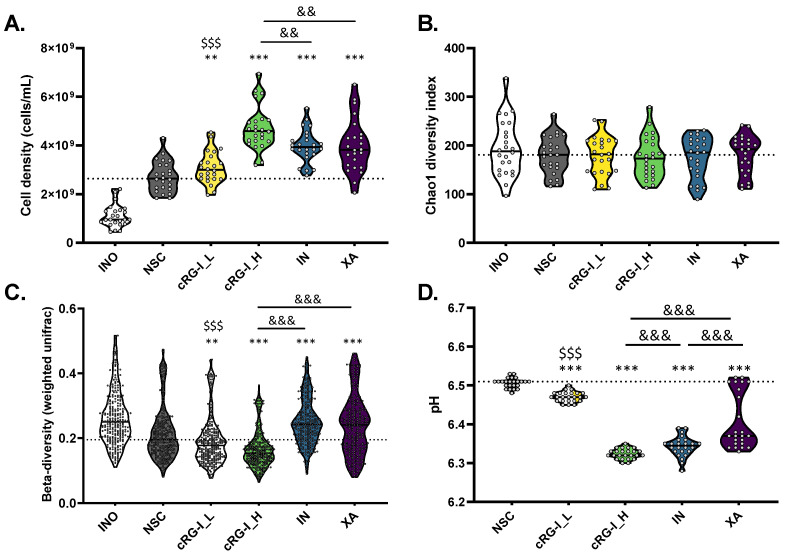
**High-dose cRG-I resulted in a marked and consistent bacterial cell density increase, along with decreases in β-diversity and pH.** Impact on (**A**) bacterial density (cells/mL), (**B**) α-diversity (Chao1 diversity index), (**C**) β-diversity (weighted Unifrac index) and (**D**) pH by treatments with cRG-I, IN, and XA at an equivalent dose of 0.3 g/d (cRG-I_L) or 1.5 g/d (cRG-I_H, IN and XA), compared to an untreated reference (NSC) for human adults (n = 24), as tested with the ex vivo SIFR^®^ technology. Statistical differences between treatments and NSC are indicated with asterisks (* (p_adjusted_ < 0.05), ** (p_adjusted_ < 0.01), or *** (p_adjusted_ < 0.001)). Further, differences between the low and high doses of cRG-I are indicated with $/$$/$$$, while differences among test compounds at an equivalent of 1.5 g/d are indicated with &/&&/&&&. SIFR = systemic intestinal fermentation research; cRG-I = carrot-derived rhamnogalacturonan; IN = inulin; XA = xanthan; NSC = no-substrate control.

The microbial growth, diversity, and pH were measured to assess overall treatment effects. First, in the untreated NSC study arm, in line with the validation study of the SIFR^®^ technology [35], marked increases in cell density were observed from 0 to 48 h, while the microbial diversity in terms of species richness was maintained at the initial level (Figure 2A,B). This preservation of in vivo derived microbiota for the entire duration of the experiment classifies the application of SIFR^®^ technology as an ex vivo study [35].

Interestingly, all test compounds (cRG-I_L, cRG-I_H, IN, and XA) increased the cell numbers significantly compared to the NSC (Figure 2A), indicating that they promoted bacterial growth. High-dose cRG-I resulted in the greatest density increase, which was significantly higher than both IN and XA (Figure 2A). Additionally, the α-diversity (i.e., a measure for species richness) was maintained for all conditions tested compared to the faecal inoculum (INO) and the NSC (Figure 2B). The β-diversity (i.e., a measure for the dissimilarity between donors within a treatment group) was significantly decreased by cRG-I in a dose-dependent manner compared to the NSC (Figure 2C), suggesting a homogenizing effect of cRG-I on the gut microbiota. In other words, the cRG-I treatment lowered the interpersonal differences among the 24 human adults. Conversely, both IN and XA increased the β-diversity compared to the NSC and cRG-I_H, suggesting that IN and XA further augmented pre-existing interpersonal differences in microbial composition. Lastly, the pH decreased for all treatments compared to the NSC, with a significantly greater decrease with cRG-I_H compared to cRG-I_L, IN, and XA (Figure 2D). There was also a marked bimodal response in the XA treated group with the microbiota of some adults showing no change in pH (nonresponders) while others showed a decrease (responders) (Figure 2D).

### 3.3. cRG-I Was Selectively Fermented by Taxa Consistently Present in the Commensal Gut Microbiota of Human Adults across Different Enterotypes

**Figure 3:**
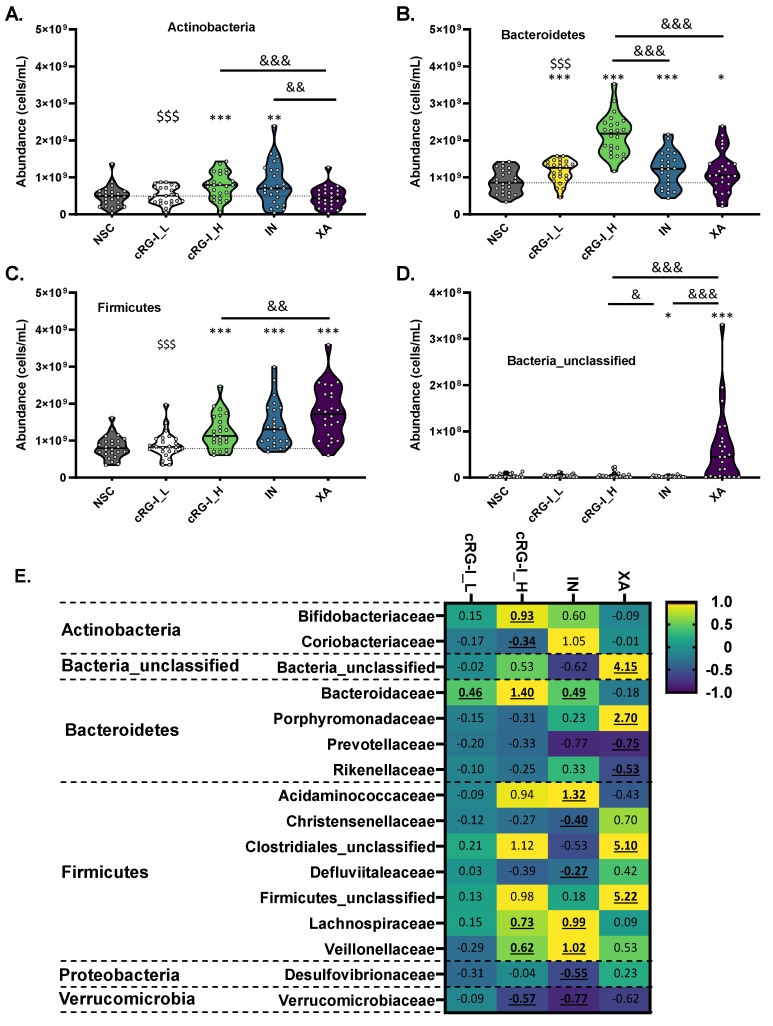
**High-dose cRG-I exerted most consistent effects on gut microbial phyla and families.** Impact on four most abundant phyla, i.e., (**A**) Actinobacteria, (**B**) Bacteroidetes, (**C**) Firmicutes, and (**D**) unclassified bacteria (cells/mL) at 48 h, upon supplementation with cRG-I, IN, and XA at an equivalent dose of 0.3 g/d (cRG-I_L) or 1.5 g/d (cRG-I_H, IN and XA), compared to an untreated reference (NSC) for human adults (n = 24), as tested with the ex vivo SIFR^®^ technology. Statistical differences between treatments and NSC are indicated with asterisks (* (p_adjusted_ < 0.05), ** (p_adjusted_ < 0.01), or *** (p_adjusted_ < 0.001)). Further, statistical differences between the low and high doses of cRG-I are indicated with $/$$/$$$, while differences among test compounds at an equivalent of 1.5 g/d are indicated with &/&&/&&&. (**E**) Heatmap showing microbial families that were significantly affected by any of the treatments, expressed as log_2_(treatment/NSC). Significant differences were indicated by bold/underlining (FDR = 0.05). SIFR = systemic intestinal fermentation research; cRG-I = carrot-derived rhamnogalacturonan; IN = inulin; XA = xanthan; NSC = no-substrate control.

Next, treatment effects on microbial composition were assessed. Given the markedly different cell densities across samples (Figure 2A), the relative abundances of taxa were normalized using cell numbers to yield more biologically relevant results, i.e., absolute abundance levels [17,39]. The first insights were obtained by a targeted analysis at the phylum level (Figure 3A–D). While cRG-I_H, IN, and XA all significantly increased Bacteroidetes and Firmicutes levels (Figure 3B,C), the families responsible for increased levels of a given phylum greatly differed between treatments (Figure 3E). For example, while the Bacteroidetes increase with cRG-I was due to a marked *Bacteroidaceae* stimulation, the increase of the same phylum with XA was due to increases in *Porphyromonadaceae*. Further, IN mildly increased both aforementioned families along with the *Rikenellaceae* family. Likewise, the *Firmicutes* increase upon cRG-I and IN supplementation was due to increases in *Acidaminococcaceae*, *Lachnospiraceae,* and *Veillonellaceae*, while XA markedly increased unclassified families. Further, cRG-I_H and IN both increased the Actinobacteria phylum (Figure 3A), while XA stimulated an unclassified Bacteria phylum (Figure 3D). The Actinobacteria increase with cRG-I was due to a marked *Bifidobacteriaceae* increase, whereas IN stimulated both *Bifidobacteriaceae* and *Coriobacteriaceae*.

Interestingly, the increased abundance of Bacteroidetes (and *Bacteroidaceae*) upon cRG-I supplementation was marked and remarkably consistent across the 24 adults. This was already significant at the five-time lower HED of 300 mg/d cRG-I (cRG-I_L).

**Figure 4:**
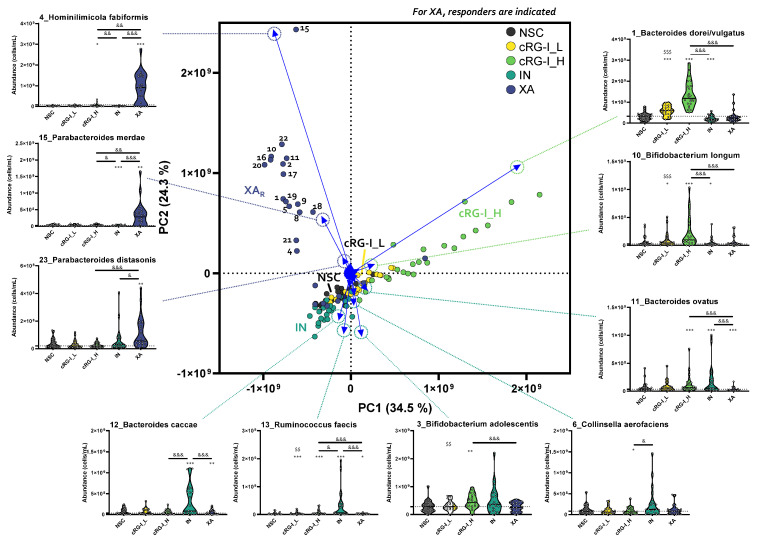
**High-dose cRG-I markedly and consistently increased OTUs related to *Bacteroides dorei/vulgatus* and *Bifidobacterium longum***. PCA biplot based on centred values of OTUs (cells/mL) along with dedicated violin plots for OTUs determining the sample distribution at 48 h, upon supplementation with cRG-I, IN, and XA at an equivalent dose of 0.3 g/d (cRG-I_L) or 1.5 g/d (cRG-I_H, IN and XA), compared to an untreated reference (NSC) for human adults (n = 24), as tested with the ex vivo SIFR^®^ technology. Statistical differences between treatments and NSC are indicated with asterisks (* (p_adjusted_ < 0.05), ** (p_adjusted_ < 0.01), or *** (p_adjusted_ < 0.001)). Further, statistical differences between the low and high doses of cRG-I are indicated with $/$$/$$$, while differences among test compounds at an equivalent of 1.5 g/d are indicated with &/&&/&&&. OTU = operational taxonomic unit; PCA = principal component analysis; cRG-I = carrot-derived rhamnogalacturonan; IN = inulin; XA = xanthan; XA_R_ = XA responders, XA_NR_ = XA nonresponders; NSC = no-substrate control; SIFR = systemic intestinal fermentation research.

The analysis at the OTU level provided a maximal resolution into how the treatments impacted microbial composition. The data were first presented in a PCA based on centred data, to focus on those species that were most markedly and consistently affected (Figure 4). This demonstrated that the most drastic changes of specific species related to the XA responder group (15/24 donors) and cRG-I_H treatments (24/24 donors). The cRG-I treatment led to marked, consistent, and dose-dependent increases of OTUs related to *Bacteroides dorei/vulgatus* and *Bifidobacterium longum* (Figure 4). Further, a narrow spectrum of XA-fermenting taxa was identified and included *Porphyromonadaceae* members (OTUs related to *Parabacteroides distasonis* and *P. merdae*) and particularly an OTU4 related to *Hominilimicola fabiformis* (Figure 4). Upon IN treatment, a broad spectrum of OTUs increased, yet given the lack of consistency across donors, these changes were not represented in the PCA that aimed to explain the largest possible variation in only two components. Taxa that were affected upon IN treatment in a highly donor-dependent manner included OTUs related to *Collinsella aerofaciens, Bifidobacterium adolescentis, Bacteroides ovatus, Bacteroides caccae,* and *Ruminococcus faecis* (Figure 4).

All significantly affected OTUs (among the 100 most abundant) were also presented in a heat map (Figure S1**)** thus providing insights in less abundant taxa. A striking observation was that cRG-I significantly increased a broad spectrum of OTUs (30 in total) belonging to *Bifidobacteriaceae* (2), *Bacteroidaceae* (4), *Acidaminococcaceae* (1), unclassified *Clostridiales* (1), *Lachnospiraceae* (18), *Ruminococcaceae* (3), and *Veillonellaceae* (1); including OTUs of health-related species such as *Bifidobacterium longum*, *Bifidobacterium adolescentis, Anaerobutyricum hallii,* and *Faecalibacterium prausnitzii*. In contrast, while a broad spectrum of OTUs tended to also increase upon IN supplementation, the large interpersonal differences of IN-related treatment effects resulted in only eight OTUs being significantly affected by the IN treatment. Finally, XA did not impact any low abundant OTUs suggesting a highly specific fermentation by the aforementioned *Hominilimicola fabiformis* and *Parabacteroides* species in donors harbouring a microbiota containing OTUs related to these species.

### 3.4. cRG-I Most Markedly and Consistently Stimulated Acetate and Propionate Production with Only Minor Increases in Gas Production

**Figure 5:**
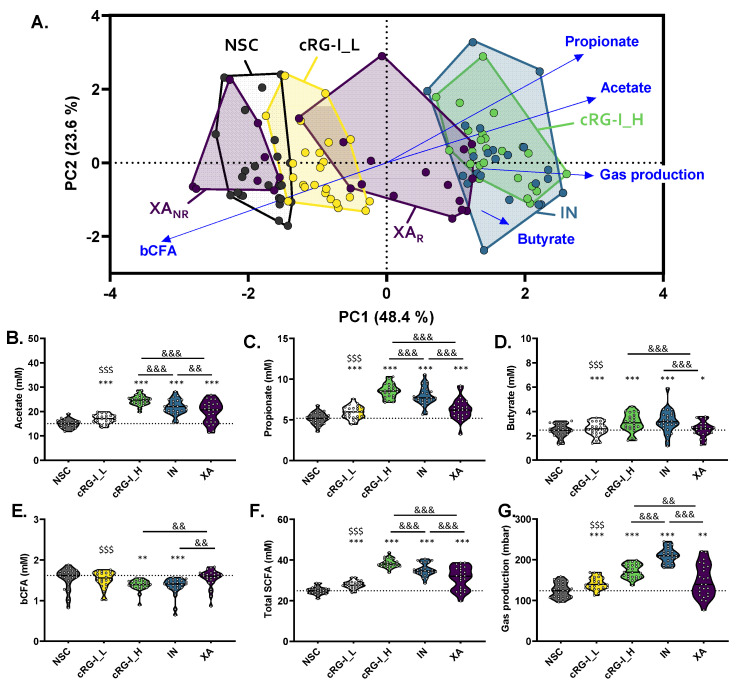
**High-dose cRG-I most strongly increased total SCFA due to a marked increase of acetate and propionate, with relatively small increases of gas production**. (**A**) PCA biplot based on standardized values of SCFA (mM) along with dedicated violin plots for (**B**) acetate, (**C**) propionate, (**D**) butyrate, (**E**) bCFA, (**F**) total SCFA, and (**G**) gas production (mbar) at 48 h, upon supplementation with cRG-I, IN, and XA at an equivalent dose of 0.3 g/d (cRG-I_L) or 1.5 g/d (cRG-I_H, IN and XA), compared to an untreated reference (NSC) for human adults (n = 24), as tested with the ex vivo SIFR^®^ technology. Statistical differences between treatments and NSC are indicated with asterisks (* (p_adjusted_ < 0.05), ** (p_adjusted_ < 0.01), or *** (p_adjusted_ < 0.001)). Further, statistical differences between the low and high doses of cRG-I are indicated with $/$$/$$$, while differences among test compounds at an equivalent of 1.5 g/d are indicated with &/&&/&&&. SCFA = short-chain fatty acids; PCA = principal component analysis; cRG-I = carrot-derived rhamnogalacturonan; IN = inulin; XA = xanthan; NSC = no-substrate control; SIFR = systemic intestinal fermentation research.

Finally, markers of microbial activity were assessed, i.e., SCFA, bCFA, and gas production. An exploratory PCA analysis revealed that IN and cRG-I_H most strongly affected these endpoints. Overall, the effect of cRG-I was again more consistent across the 24 human adults as shown by the smaller surface area of data clustering for cRG-I compared to IN (Figure 5A). Further, XA-treated samples again displayed a bimodal distribution, with XA responders (XA_R_) and XA non responders (XA_NR_) positioning in the middle and to the left, respectively (Figure 5A).

All treatment conditions significantly increased acetate, propionate, and butyrate compared to NSC, with greater increases in acetate and propionate in cRG-I_H compared to other treatments (Figure 5B–D). While both substrates were dosed at 1.5 g/d, cRG-I resulted in significantly higher acetate (+40%) and propionate (+22%) levels compared to IN. Further, only cRG-I_H and IN significantly decreased bCFA compared to NSC (Figure 5E). Total SCFA were significantly increased by all treatments compared to NSC, with a higher increase in the cRG-I_H-treated samples compared to other compounds (Figure 5F) (+32% compared to IN). In XA-treated samples, a bimodal response profile was again observed for total SCFA with some samples showing no change (Figure 5F). Gas production also increased in all treatments compared to NSC, with a higher increase in the IN-treated samples compared to other compound treatments (Figure 5G). Despite the marked SCFA production with cRG-I_H, gas production was remarkably lower compared to IN (−44%) (Figure 5G).

When examining the coefficient of variations (CVs) for each analyte, XA had the highest CVs for total SCFA, acetate, and propionate as well as gas production compared to other test compounds (Figure S2). The IN treatment had the highest CVs for butyrate and for bCFA, while the high and low doses of cRG-I had the lowest CVs for SCFA and bCFA (Figure S2). This indicated that IN and XA led to higher interpersonal differences in microbial metabolite production, while high-dose cRG-I led to lower interpersonal differences even when compared to NSC for most parameters. In other words, cRG-I resulted in predictable effects on microbial metabolite production.

### 3.5. Specific OTUs Correlated with Specific Metabolites upon cRG-I Supplementation

**Figure 6:**
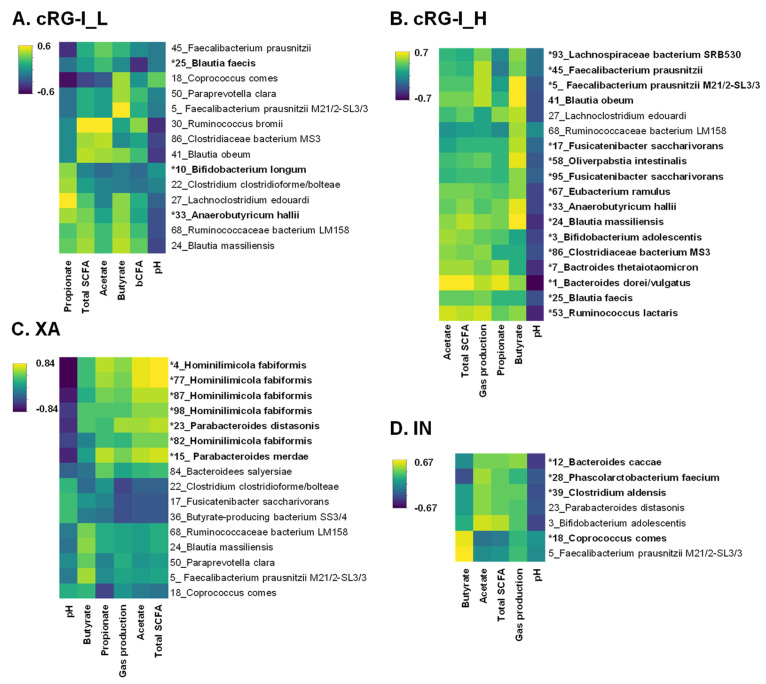
**High-dose cRG-I exerted consistent stimulatory effects on a range of OTUs, which resulted in marked correlations between specific fundamental fermentation parameters and these OTUs**. Heatmap showing correlations based on a rCCA between fermentation parameters (pH, gas production, and SCFA) and OTUs that were significantly affected (FDR = 0.05) by any of the treatments, i.e., (**A**) cRG-I_L, (**B**) cRG-I_H, (**C**) XA, and (**D**) IN for human adults (n = 24), as tested with the ex vivo SIFR^®^ technology. The OTUs that were significantly increased by a given treatment are preceded by an asterisk in the plot of this treatment. OTU = operational taxonomic unit; rCCA = regularized canonical correlation analysis; SCFA = short-chain fatty acids; cRG-I = carrot-derived rhamnogalacturonan; IN = inulin; XA = xanthan; SIFR = systemic intestinal fermentation research.

To understand the relationship between changes in fermentation parameters and changes in microbial composition, correlation analyses were performed between fermentation parameters and the 49 OTUs that were significantly affected by at least one treatment (Figure 6). Interestingly, for the low and particularly the high dose of cRG-I (Figure 6A,B), many of the OTUs significantly stimulated by cRG-I (indicated with an asterisk) related with specific SCFA, suggesting the involvement of specific species during cRG-I fermentation. In line with the metabolic ability of these taxa, OTUs related to *B. dorei/vulgatus* and *B. thetaiotaomicron* correlated with acetate and propionate, while butyrate production was associated with the presence of OTUs related to *Blautia* species (OTU24/41), *A. hallii* (OTU33), and *F. prausnitzii* (OTU5). Further, for XA, marked correlations were established for XA-fermenting taxa: while OTU4/77 correlated with acetate production, the OTU related to *P. merdae* more strongly correlated with propionate (Figure 6C). Finally, for IN, less correlations were observed as these were obscured by the high interpersonal differences observed upon IN treatment (Figure 6D). Nevertheless, interesting correlations of acetate with *B. adolescentis* and butyrate with *C. comes* and *F. prausnitzii* were established, suggesting that these species are key drivers for pathways producing these SCFA upon IN treatment (Figure 6D).

## 4. Discussion

The high-throughput, ex vivo SIFR^®^ technology enabled the testing of the impact of supplementation with different fibres on the faecal microbiota of 24 human adults in parallel. Such a large number is unprecedented in preclinical in vitro study designs. This was of key importance to not only ensure representative findings but to also accurately evaluate the selectivity by which various fibres impact the human gut microbiota. The biorelevance of the interpersonal differences among the 24 test subjects also followed from the observation that they were driven by differential levels of *Prevotellaceae*, *Bacteroidaceae,* and/or *Ruminococcaceae*, in line with the well-known concept of enterotypes [16,17,18,19]. Overall, the key finding of the study was that cRG-I treatment lowered interpersonal differences in microbial composition and metabolite production due to the selective stimulation of taxa that were consistently present in the gut microbiome of human adults. This contrasted with IN and XA, which enhanced interpersonal differences. The remarkable homogenization effect of cRG-I highlighted in the current SIFR^®^ experiment was recently also observed in a randomized placebo-controlled clinical trial, where treatments with 0.3 g/d and 1.5 g of cRG-I/d were shown to significantly lower interindividual microbiota variability measured as the between-subject β-diversity after 8 weeks of intervention (manuscript in preparation). Of note, the striking decrease in interindividual variation had earlier been observed in a mouse model of enteric infection as well (manuscript in preparation). This confirms the remarkable homogenization effect of cRG-I, the coherence of the effect even across models and host species, and further confirms the predictivity of the SIFR^®^ technology for the impact of fibre supplementation in (human) intervention studies [35].

The specific taxa that increased upon cRG-I treatment included, amongst others, acetate/propionate-producing *Bacteroidaceae* (*B. dorei/vulgatus* and *B. thetaiotaomicron)* [10], acetate-producing *Bifidobacteriaceae* (*B. longum* and *B. adolescentis*) [40], and butyrate-producing species such as *Anaerobutyricum hallii* [41,42], *Blautia* sp. [43,44], and *Faecalibacterium prausnitzii* [45]. The marked correlation between these species and said SCFA is in line with their respective metabolic capabilities. It further emphasizes the advantage of including a large number of subjects as this is key to establishing correlations between metabolites and bacterial species and thus shedding light on the mechanistic effect of cRG-I on the gut microbiota. While this is already possible with 6 test subjects [35], correlations are even stronger when working with 24 test subjects. The two most profoundly increased OTUs were those related to *B. dorei/vulgatus* and *B. longum,* suggesting that these species are likely keystone species for cRG-I fermentation, as previously suggested [27,28]. While *Bacteroides* species are generally known to possess a broad range of carbohydrate-active enzymes (CAZymes) within their polysaccharide utilization loci (PUL) [46,47], *B. dorei/vulgatus* seems to be the most competitive amongst *Bacteroides* species to ferment cRG-I. The initial fermentation of large cRG-I polymers by *Bacteroides* species likely releases cRG-I fragments that are then consumed by *Bifidobacteriaceae*, known degraders of arabinans and galactans, the main side chains of cRG-I [48,49]. Such cooperative mechanisms between *Bacteroides* and *Bifidobacterium* species in the fermentation of complex polymers have been described before [50]. Moreover, the current study suggests that this initial step feeds a trophic network involving a spectrum of phylogenetically diverse species that all benefited from cRG-I treatment. Indeed, 30 OTUs not only belonging to *Bifidobacteriaceae* and *Bacteroidaceae*, but also to *Acidaminococcaceae*, unclassified *Clostridiales*, *Ruminococcaceae*, *Veillonellaceae,* and particularly *Lachnospiraceae,* all significantly increased upon cRG-I treatment. As demonstrated for other complex polysaccharides [51], the high complexity of the cRG-I chemical structure is likely responsible for supporting the growth of such a broad range of taxa. The consistency of how these taxa were boosted among the 24 human adults was, however, highly remarkable and resulted in robust effects on metabolite production as well. This striking consistency could result in predicable outcomes of cRG-I interventions in healthy subjects despite differences in gut microbiota composition. In line with the recently proposed classification of fibres based on their “specificity” [21,22], cRG-I displays properties of a medium-high specificity fibre, thus reliably increasing the abundance of beneficial bacterial species commonly found in the gut microbiota of healthy adults.

While XA also significantly decreased pH and boosted SCFA production (mostly acetate and to a lesser extent, propionate), these changes were less pronounced compared to cRG-I, given that 9 of the 24 donors were nonfermenters to XA treatment. XA fermentation related to markedly increased levels of a very narrow spectrum of OTUs related to *Parabacteroides merdae*, *Parabacteroides distasonis,* and particularly *Hominilimicola fabiformis,* a species recently isolated by Afrizal et al. via single-cell dispensing [52]. The absence of *H. fabiformis* was characteristic of the microbiota of nonfermenters. This is in line with recent findings of Ostrowski et al. (2022) [26], who demonstrated an uncultured *Ruminococcaceae* species (*R.* UCG13) to be the keystone degrader of the xanthan gum backbone via a novel glycoside hydrolase family 5 (GH5) enzyme. Interestingly, the 16S rRNA gene sequence of *R.* UCG13 revealed a >99% sequence similarity with *H. fabiformis* (M. Ostrowski, personal communication, 4 April 2023). While unable to ferment the high molecular weight polymer, *Bacteroides/Parabacteroides* species were shown capable of consuming oligosaccharides generated by the keystone *H. fabiformis* species. As XA thus classifies as a very high specificity fibre, it depends on the presence of keystone species capable of initiating its colonic fermentation to potentially lead to health benefits. Very high specificity fibres solely fermented by keystone species that are not consistently present among the gut microbiota of individuals (as is the case for XA) may thus lead to poorly predictable outcomes in human studies, as they increase the overall interpersonal differences upon supplementation.

IN treatment also markedly enhanced the intersubject β-diversity. This resulted from donor-dependent increases of a variety of OTUs related among others to *Collinsella aerofaciens*, *Bifidobacterium adolescentis*, *Bacteroides ovatus*, *Bacteroides caccae*, *Ruminococcus faecis,* and *Coprococcus comes*. Another example of the low specificity of IN was the marked correlation of the increase in *Faecalibacterium prausnitzii* and butyrate levels upon IN treatment in some but not all donors. Such a strong donor-dependent microbiota modulation is in agreement with recent studies that describe IN as a low specificity fibre used by a wide array of gut microbes [24,25,35]. As a remark, the stimulation of *Bifidobacteriaceae* is the most pronounced microbiota change commonly induced by IN in clinical studies [53]. In the clinical study performed by Vandeputte et al., IN was dosed at 12 g/d, possibly leading to a profound colonic pH decrease. High-fibre doses do not only impact the gut microbiota but also change the gut environment as was demonstrated by marked luminal pH decreases when dosing high amounts of IN to humanized rats [54]. Such a lower pH likely explains the stronger effects of IN dosed at levels above 10 g/d on acid-tolerant *Bifidobacteriaceae* in contrast to *Bacteroides* species that thrive better at higher pH [55]. The low dose simulated during the current study (1.5 g/d) explains the milder effects on *Bifidobacteriaceae.* Overall, while low-specificity fibres (such as IN) offer the advantage that there are no or less nonfermenters, the outcomes of such interventions are less predictable as the baseline interindividual differences will be maintained or even enhanced.

As described above, inulin and cRG-I both stimulate *Bifidobacterium* species, albeit in a specific manner. The health benefits attributed to *Bifidobacterium* species are numerous and include antipathogenic effects, immune modulation, the prevention of gut disorders, and the production of beneficial metabolites and vitamins [56,57,58,59]. High counts of bifidobacteria have frequently been correlated to health, which has led to the development of *B. longum* and *B. adolescentis* strains as probiotics [60]. While inulin is easily digested by numerous gut commensals, the cRG-I fermentation necessitates the cooperation between *Bacteroides* and *Bifidobacterium spp.* acting as primary and secondary degraders, respectively, further promoting the cross-feeding of key anti-inflammatory species such as *F*. *prausnitzii* [61] and *A. hallii* [62]. *Bacteroides* spp., on the other hand, have been associated with health or diseased situations depending on the species, their intestinal location, and the host health status [63,64,65] but play an important role in feeding the gut metabolic network.

When focusing on changes in fundamental fermentation parameters, cRG-I resulted in significantly higher acetate (+40%), propionate (+22%), and thus also total SCFA levels (+32%) compared to IN, both dosed at an equivalent of 1.5 g/d. Nevertheless, gas production was significantly lower for cRG-I compared to IN (−44%). A strong gas production upon intake of IN (or other fructans such as FOS) due to a rapid colonic fermentation has been observed before and could result in limited tolerance at high doses [66,67]. Interestingly, cRG-I thus combines the property of leading to a pronounced production of SCFA [8,9] combined with a low gas production that should translate in a better tolerability.

## 5. Conclusions

In conclusion, unlike IN, cRG-I and XA were shown to be high and very high specificity fibres, respectively, possessing chemical characteristics that likely allow them to be utilized by a selected or only a narrow group of intestinal commensals, thus supporting the need for developing more selective or targeted gut microbiota modulators with more predictable outcomes [14,24,25]. The current study also pointed out that high-specificity fibres could also be overly specific, as is the case for XA, for which a considerable number of test subjects were not responding to treatment due to the absence of the highly specialized keystone XA degraders. In contrast, the keystone species involved in cRG-I fermentation were consistently present among human adults regardless of the enterotype, so that cRG-I was fermented by all donors tested, resulting in a remarkably robust impact on both microbiota composition and function (as measured by fermentation parameters). This suggests that cRG-I is a remarkable medium-high-specificity fibre leading to the targeted stimulation of specific *Bifidobacterium* and *Bacteroides* spp. and this could lead to consistent health outcomes in humans.

## Data Availability

The datasets generated during and/or analyzed during the current study are available from the corresponding author upon reasonable request.

## Supplementary Materials

The following supporting information can be downloaded at: https://www.mdpi.com/article/10.3390/nu15092090/s1, Figure S1. High-dose cRG-I exerted most consistent effects on OTUs mostly belonging to the *Bifidobacteriaceae*, *Bacteroidaceae*, *Lachnospiraceae,* and *Ruminococcaceae*, Figure S2. High-dose cRG-I resulted in low interpersonal differences in terms of SCFA and bCFA pro-duction, while IN (low specificity) and XA (very high specificity resulting in responders and nonresponders) increased interpersonal differences.
