# Supplementary material for: Carrot RG-I Reduces Interindividual Differences between 24 Adults through Consistent Effects on Gut Microbiota Composition and Function Ex Vivo
Source: Nutrients. 2023 Apr 26;15(9):2090. doi: 10.3390/nu15092090 (PMC10180869; doi:10.3390/nu15092090)
Supplement: Supplementary file 1 [file nutrients-15-02090-s001.zip › nutrients-2352537-supplementary.pdf]

Supplementary Materials

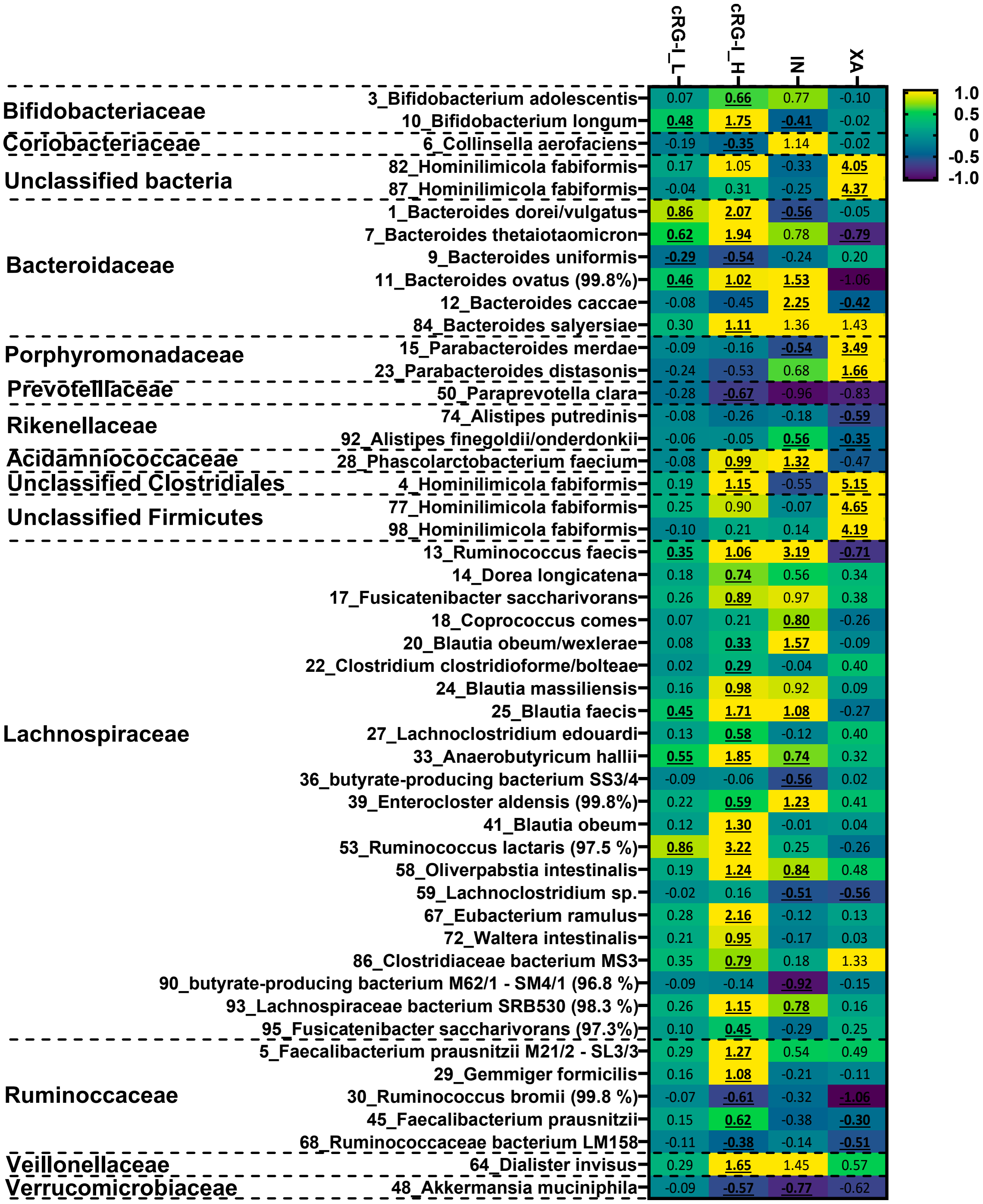

Figure S1. High dose cRG-I exerted most consistent effects on OTUs mostly belonging to the *Bifidobacteriaceae, Bacteroidaceae, Lachnospiraceae* and *Ruminococcaceae.* Heatmap showing OTUs (related to specific species) that were significantly affected (FDR = 0.05) by any of the treatments, expressed as log_2(treatment/NSC) at 48h upon treatment with cRG-I, IN and XA at an equivalent dose of 0.3g/d (cRG-I_L) or 1.5g/d (cRG-I_H, IN and XA), compared to an untreated reference (NSC) for human adults (n = 24), as tested with the *ex vivo* SIFR® technology. Significant differences were indicated by bold/underlining (FDR = 0.05). OTU = operational taxonomic unit; FDR = false discovery rate; SIFR = systemic intestinal fermentation research; cRG-I = carrot-derived rhamnogalacturonan; IN = inulin; XA = xanthan; NSC = no substrate control.

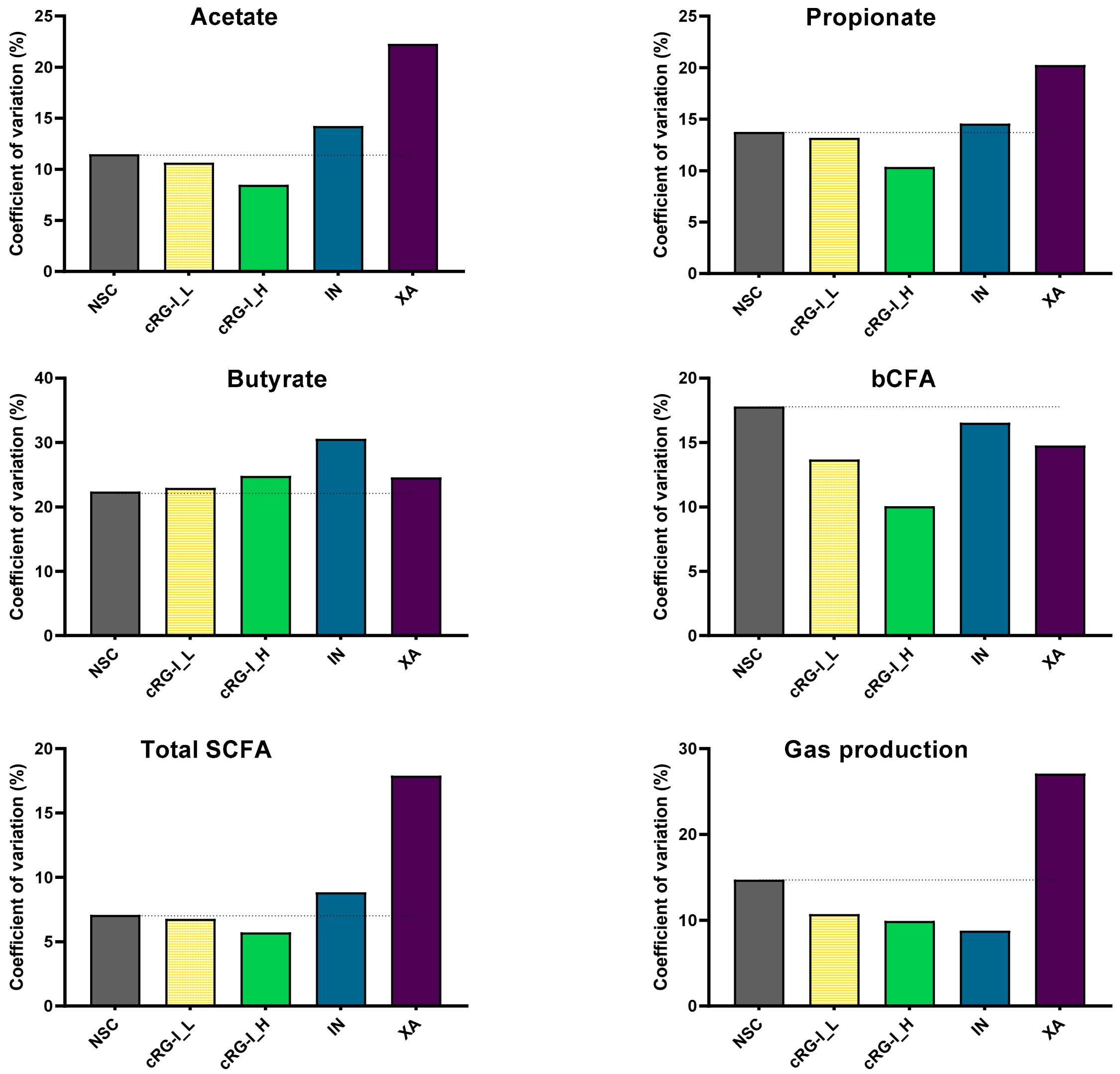

Figure S2. High dose cRG-I resulted in low interpersonal differences in terms of SCFA and bCFA production. Coefficient of variation (= standard deviation/average; %) across the 24 human adults for values of acetate (A), propionate (B), butyrate (C), bCFA (D), total SCFA (E) and gas production (H), as tested via the SIFR® technology platform, for carrot-derived rhamnogalacturonan I (cRG-I), inulin (IN) and xanthan, dosed at an equivalent of 0.3g/d (cRG-I_L) or 1.5g/d (cRG-I_H, IN and XA), as compared to an untreated blank. Samples were collected after 48h of simulated colonic incubations. SCFA = short-chain fatty acids; cRG-I = carrot-derived rhamnogalacturonan; IN = inulin; XA = xanthan; NSC = no substrate control; SIFR = systemic intestinal fermentation research.
